# Supplementary material for: Distinct Bacterial Communities in São Jorge Cheese with Protected Designation of Origin (PDO)
Source: Foods. 2023 Feb 26;12(5):990. doi: 10.3390/foods12050990 (PMC10000650; doi:10.3390/foods12050990)
Supplement: Supplementary file 1 [file foods-12-00990-s001.zip › foods-2219685-supplementary.pdf]

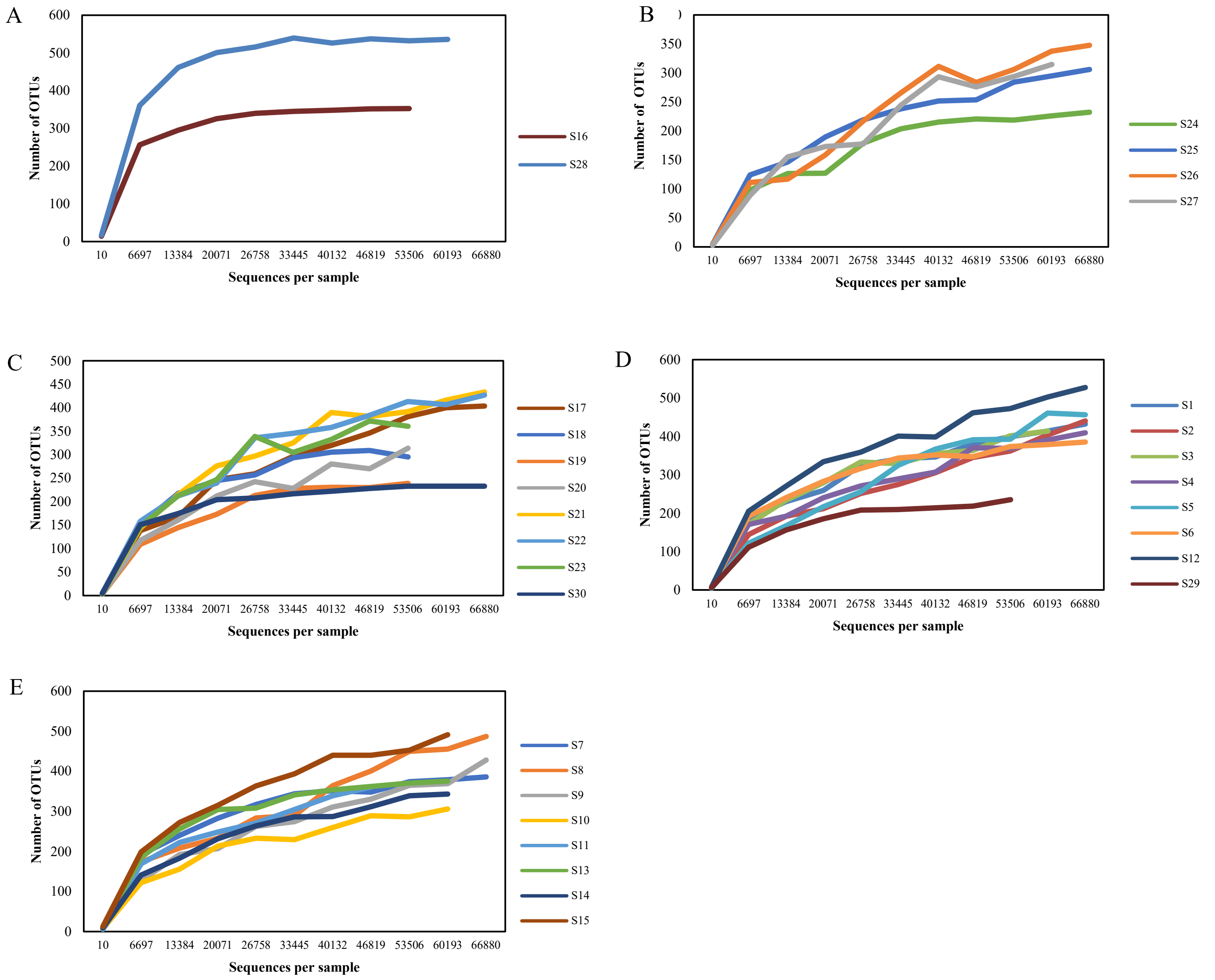

Figure S1. Rarefaction curves of the variable region of the 16S rRNA sequences of samples obtained during the production of São Jorge cheese. A) Milk (samples S16 and S28); B) Whey used as a starter culture in the production of São Jorge cheese (samples S24-27); C) Curd (samples S17-S23, S30); D) Non-PDO cheese (samples S1-S6, S12, S29). B) PDO cheese (samples S7-S11, S13-S15).
